# The NAC Transcription Factors *CjNAC43* and *CjNAC54* Act as Positive Regulators of Leaf Senescence in *Clerodendrum japonicum*

**DOI:** 10.3390/ijms27010133

**Published:** 2025-12-22

**Authors:** Yanwen Deng, Congcong Wang, Rutao Huang, Lingye Su, Chunmei He, Mingfeng Xu, Hongfeng Wang

**Affiliations:** Guangdong Provincial Key Laboratory of Silviculture, Protection and Utilization, Guangdong Academy of Forestry, Guangzhou 510520, China

**Keywords:** *Clerodendrum japonicum*, NAC transcription factors, leaf senescence, ABA signaling, dark-induced senescence, VIGS, functional characterization

## Abstract

Leaf senescence is a crucial developmental process in plants, and is tightly regulated by transcription factors such as NAC family members. However, the functions of NAC genes in the leaf senescence of the medicinal and ornamental plant *Clerodendrum japonicum* remain largely uncharacterized. In this study, we performed the transcriptome sequencing of mature and early-senescent leaves in *C. japonicum*. We screened candidate NAC genes and validated their expression patterns using quantitative real-time PCR (qRT-PCR). The functions of *CjNAC43* and *CjNAC54* were characterized through heterologous overexpression in *Arabidopsis thaliana* and Virus-Induced Gene Silencing (VIGS) in *C. japonicum*. We further investigated their roles in abscisic acid (ABA)- and dark-induced senescence. Our findings revealed that transcriptomic analysis identified 522 differentially expressed genes, including nine NAC members. *CjNAC43* and *CjNAC54* exhibited significantly upregulated expression during the critical senescence phase (90–130 days). Overexpression of either gene in *A. thaliana* accelerated leaf senescence, up-regulated senescence-associated genes (*SAG*s), and reduced chlorophyll content. Conversely, silencing *CjNAC43* or *CjNAC54* in *C. japonicum* delayed senescence. Both genes enhanced the plant’s sensitivity to ABA and darkness, leading to accelerated senescence under these stresses. In conclusion, our results demonstrate that *CjNAC43* and *CjNAC54* function as positive regulators of leaf senescence in *C. japonicum*, partly by mediating ABA and dark signaling pathways.

## 1. Introduction

Leaves are the primary organs responsible for photosynthesis in plants, converting light energy into chemical energy and thereby supplying the essential resources required for growth and development [1]. As the terminal phase of leaf development, leaf senescence represents a highly regulated and irreversible developmental process [2]. This stage is typically manifested by chlorophyll degradation and progressive loss of leaf functionality [3], serving to minimize energy expenditure while maintaining fundamental physiological activities [4]. This process allows for the remobilization of nutrients from aging leaves to developing tissues such as young leaves, fruits, and seeds. During senescence, photosynthetic efficiency declines markedly, resulting in reduced energy acquisition and diminished carbon assimilation capacity [5]. Concurrently, extensive degradation of cellular constituents—including proteins, lipids, nucleic acids, and chlorophyll-occurs. These macromolecules are subsequently mobilized through vascular tissues to growing points, reproductive organs, and seeds [5], thereby facilitating the redistribution of nutrients from source tissues to sink organs [6]. Such nutrient remobilization not only enhances resource-use efficiency [7], but also ensures the successful completion of the reproductive phase [8]. Consequently, leaf senescence exerts a profound influence on plant growth and development and simultaneously serves as a crucial adaptive strategy, enabling plants to optimize resource allocation under varying environmental conditions [9]. Moreover, this process holds significant implications for the yield of medicinal plants and the maintenance of ornamental stability in urban greening species [10].

Leaf senescence is a complex and highly orchestrated biological process [5], governed by the integration of multi-level signals that encompass both hormonal regulation and environmental cues [11]. Among the key phytohormones, abscisic acid (ABA) and ethylene (ET) have emerged as central regulators, promoting the initiation and progression of senescence by modulating transcription factor activity and downstream gene expression [12,13]. In parallel, external environmental stresses—such as prolonged darkness, drought, or elevated temperatures—can markedly accelerate the onset of leaf senescence, highlighting the interplay between intrinsic and extrinsic factors in this process [14,15]. Among these, darkness is a potent inducer of leaf senescence, often used experimentally to dissect the underlying molecular mechanisms. One of the earliest and most visually discernible events during leaf senescence is chlorophyll degradation [4,12,16]. This biochemical process is tightly controlled, involving a coordinated network of enzymes and regulatory proteins. For example, STAY-GREEN 1 (SGR1) facilitates the dissociation of chloroplast antenna proteins from the thylakoid membrane, thereby triggering the chlorophyll degradation pathway [16,17], whereas PHEOPHORBIDE A OXYGENASE (PAO) catalyzes the rate-limiting step in chlorophyll catabolism, representing a crucial checkpoint in leaf yellowing [18]. In addition, classical senescence-associated genes (SAGs), such as SAG12 and SAG113, contribute to the degradation of cellular components, regulation of endogenous ABA levels, and maintenance of water homeostasis [12,19]. Moreover, ACTIN 2 (ACT2), a core element of the cytoskeletal microfilaments, supports structural stability throughout the senescence process [20]. These senescence-related genes serve as reliable molecular markers, allowing precise evaluation of leaf aging stages and cellular responses to various stimuli. Consequently, they have become indispensable tools in functional genomics studies and molecular analyses of leaf senescence across diverse plant species and experimental conditions [21].

Among the myriad transcription factors (TFs) governing leaf senescence, the NAC (NAM/ATAF/CUC) transcription factor family stands out as one of the most pivotal groups [22,23]. NAC proteins are not only abundant but also exhibit remarkable functional diversity across plant species. To date, genome-wide analyses have identified 138 NAC genes in *A. thaliana* [24], 151 in rice (*Oryza sativa*) [25], and 152 in maize (*Zea mays*) [26]. Importantly, several NAC genes have been characterized as positive regulators of senescence. Prominent examples include AtNAP (ANAC029) and AtORE1 (ANAC092) in *A. thaliana* [27,28], OsNAP (ONAC), OsNAC103, and ONAC011 in rice [29,30] and ZmNAP and ZmNAC126 in maize [26,31]. These key NAC transcription factors occupy upstream positions within the senescence regulatory network and are capable of directly or indirectly modulating the expression of chlorophyll catabolic genes, such as *STAY-GREEN* 1 (*SGR*1), *NON-YELLOW COLORING* 1 (*NYC*1), and *PHEOPHORBIDE A OXYGENASE* (*PAO*) [28,32], as well as central nodes in the abscisic acid (ABA) and ethylene (ET) signaling pathways, including *ABSCISIC ACID INSENSITIVE* 5 (*ABI*5) and *SENESCENCE-ASSOCIATED GENE* 113 (*SAG*113) [19,33]. Through such coordinated regulation, NAC proteins synergistically orchestrate senescence-associated processes, including leaf yellowing and programmed cell death. Accordingly, the NAC family functions as both an integrator and amplifier of multiple signaling pathways, acting as a core regulator that initiates the leaf senescence program [12]. Importantly, the regulatory functions of NAC TFs in integrating developmental cues with environmental stress responses to modulate senescence have also been demonstrated in key agronomic species, such as sunflower and wheat [34,35,36]. Although NAC genes have been extensively investigated in model herbaceous species, their molecular roles in woody plants remain comparatively underexplored [37]. Previous studies have systematically examined NAC function in woody species such as poplar [38] and grape [39]. Nevertheless, direct evidence linking NAC transcription factors to leaf senescence in the genus *Clerodendrum* is still exceedingly scarce.

Despite the well-established roles of NAC TFs in leaf senescence of model plants, their functions in woody species, particularly in non-model ornamental plants like *C. japonicum*, are less explored. *Clerodendrum japonicum* is a plant of substantial medicinal potential and considerable ornamental value, distinguished by its striking red inflorescences and visually appealing fruit [40]. Bioactive extracts from this species have been reported to exhibit anti-inflammatory and antibacterial activities, highlighting its pharmacological significance [41,42]. To date, research on *C. japonicum* has predominantly concentrated on the characterization of chemical constituents, pharmacological evaluation, and elucidation of biosynthetic pathways [41]. More recently, transcriptomic analyses have begun to shed light on the molecular mechanisms underlying leaf senescence in this species [43]. However, functional validation of candidate genes remains largely unexplored.

Therefore, the molecular mechanisms, especially the regulatory roles of NAC TFs, controlling its leaf senescence are still elusive. Given that leaf senescence directly impacts the yield of bioactive compounds in medicinal plants and the longevity of ornamental display, elucidating its regulatory network in *C. japonicum* has both economic and horticultural relevance. As central regulators of senescence in model plants, NAC TFs represent key candidates for such investigation. Their functional characterization in this non-model woody species would not only fill a knowledge gap but also provide a molecular basis for potential strategies to modulate senescence, such as breeding for delayed leaf yellowing to extend its ornamental period. Therefore, understanding the roles of NAC transcription factors in regulating leaf senescence in *C. japonicum* is therefore of critical importance. In this study, Among the differentially expressed NAC genes, *CjNAC43* and *CjNAC54* were selected for further investigation. Phylogenetic analysis indicated that CjNAC43 is an ortholog of *Arabidopsis* ANAC002/ATAF1, a well-characterized positive regulator of leaf senescence that integrates aging and stress signals [44]. CjNAC54 clusters with the *Arabidopsis* senescence-associated subgroup containing ANAC097, ANAC084, and ANAC104, members of which are implicated in reactive oxygen species homeostasis and cell death [45]. The specific and sustained upregulation of *CjNAC43* and *CjNAC54* during the critical senescence window (90–130 days), as confirmed by our qRT-PCR analysis, marked them as prime functional candidates. Therefore, the primary purpose of this study was to determine whether *CjNAC43* and *CjNAC54* function as regulators of leaf senescence in *C. japonicum*. To this end, we characterized the functions of *CjNAC43* and *CjNAC54* using heterologous overexpression in *A. thaliana*, combined with stress treatments and virus-induced gene silencing (VIGS) in *C. japonicum*. Such studies not only provide a theoretical basis for the development of cultivars with delayed senescence but also help bridge the current knowledge gap regarding the molecular control of leaf senescence in woody plants.

## 2. Results

### 2.1. Transcriptomic Analysis and Selection of Differentially Expressed Genes

**Figure 1:**
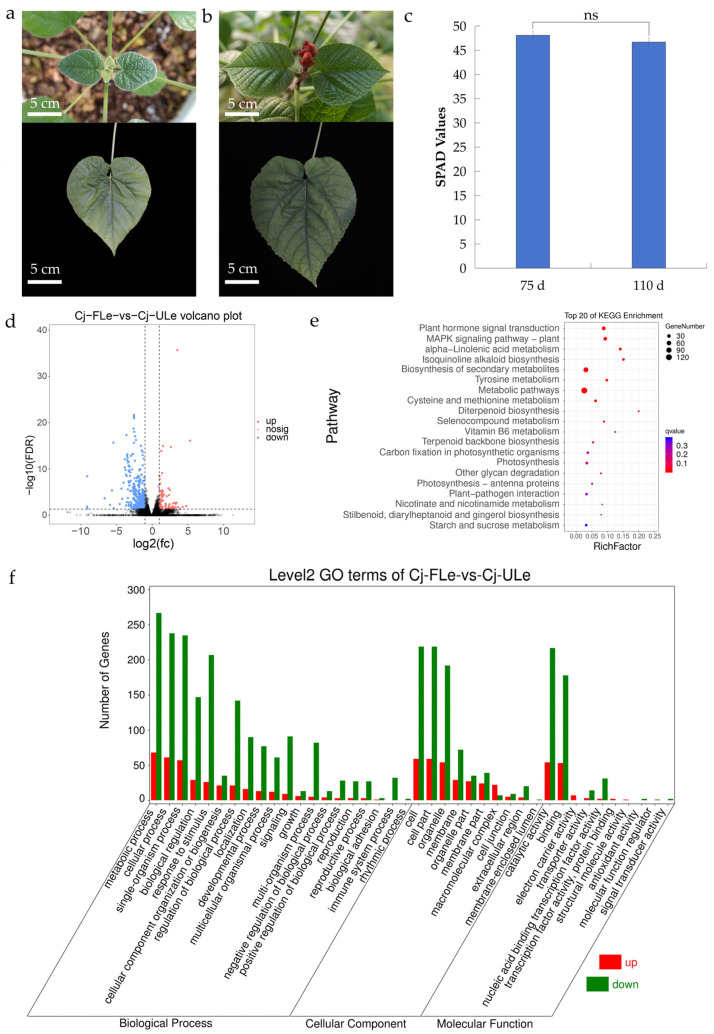
Transcriptomic profiling of leaf senescence in *C. japonicum*. (**a**) Phenotype of a representative mature leaf sampled from the 3rd to 5th nodal position at 75 days after leaf emergence (ULe stage), showing no flower bud formation and relatively green color. (**b**) Phenotype of an early-senescent leaf from the same nodal position (3rd to 5th) of a different plant cohort at 110 days after leaf emergence (FLe stage), showing visible flower bud development and initial yellowing. To ensure comparability, leaves from equivalent developmental positions were used for all analyses. (**c**) Leaf chlorophyll content measured as SPAD values at 75 and 110 days. Data are mean ± SD (*n* ≥ 3). No significant (ns) differences were observed (*p* > 0.05). Data are mean ± SD (*n* ≥ 3). No significant differences were observed between 75 d and 110 d (*p* > 0.05). (**d**) Volcano plot of differentially expressed genes (DEGs) between ULe and FLe stages. Red dots represent up-regulated genes (FDR < 0.05 and log_2_FC > 1), blue dots represent down-regulated genes (FDR < 0.05 and log_2_FC < −1), and gray dots represent non-DEGs. (**e**) Top 20 enriched KEGG pathways of DEGs between ULe and FLe leaves. (**f**) Level 2 Gene Ontology (GO) enrichment analysis of DEGs between ULe and FLe leaves, categorized into Biological Process, Cellular Component, and Molecular Function.

To elucidate the regulatory mechanisms underlying leaf senescence in *C. japonicum*, we sampled leaves from the 3rd to 5th nodal positions to ensure developmental uniformity. RNA-Seq was performed to generate gene expression profiles at two developmental stages: the mature, unflowered leaf (ULe) stage (75 days after leaf emergence; Figure 1a,c) and the early flowering leaf (FLe) stage, corresponding to the onset of senescence (110 days after leaf emergence; Figure 1b,c). No significant differences in leaf chlorophyll content, estimated using a Soil Plant Analysis Development (SPAD) meter, were observed between day 75 and day 110 (Figure 1c). After quality control and removal of low-quality reads, the average proportion of clean reads in the ULe samples was 99.87%, with only 0.03% identified as low-quality data. These results confirm the high quality of the sequencing data and the reliability of subsequent analyses. For the FLe samples, the average clean read ratio and low-quality read ratio were 99.83% and 0.05%, respectively (Table S2). Base composition analysis revealed that, after quality filtering, the average GC (Guanine-cytosine) content of the ULe and FLe samples was 48.48% and 47.77%, respectively (Table S2). Sequence alignment against the full-length *C. japonicum* transcriptome reference showed mapping rates of 86.71% for ULe and 85.63% for FLe samples (Table S3). These results confirm the high quality of the sequencing data and the reliability of subsequent analyses.

Based on the FPKM (Fragments Per Kilobase of exon model per Million mapped fragments) values of the ULe and FLe samples, a total of 522 genes were identified as significantly differentially expressed (Figure 1d), using a threshold of FDR < 0.05 and |log_2_FC| > 1. Among these, 105 genes were upregulated and 417 were downregulated (Tables S4 and S5). Upregulated genes were primarily enriched in key biological processes, including protein synthesis, metal ion transport, carbohydrate metabolism, photosynthesis, and adenosine triphosphate (ATP) synthesis. Notably, the enhanced expression of genes related to photosynthesis and protein synthesis may contribute to the adaptation of *C. japonicum* leaves to reduced photosynthetic activity and environmental stress during early senescence (Table S4). In contrast, the downregulated genes were mainly associated with metabolic regulation, heavy metal stress responses, transcriptional regulation, and fatty acid metabolism, suggesting that these pathways play crucial roles in modulating the environmental adaptability of leaves during senescence (Table S5).

To identify DEGs (Differential expressed genes) involved in the regulation of biological functions during leaf senescence, KEGG (Kyoto Encyclopedia of Genes and Genomes) and GO (Gene Ontology) enrichment analyses were performed (Figure 1e,f). The KEGG enrichment analysis (Figure 1e) revealed that DEGs were significantly enriched in pathways including plant hormone signal transduction, MAPK signaling pathway, and biosynthesis of secondary metabolites, suggesting active involvement of hormonal and stress-response networks during the transition to senescence. These findings suggest that hormonal regulation of growth and development, as well as responses to environmental stimuli, are actively involved in the transition from the Unflowered leaf (ULe)and Flowering leaf (FLe) stages in *C. japonicum*, providing important insights into the functional and metabolic regulatory mechanisms of leaf senescence. GO enrichment analysis (Figure 1f) indicated that within the biological process category, DEGs were significantly enriched in metabolic and cellular processes, reflecting substantial functional changes associated with senescence. In the cellular component category, most DEGs were enriched in “cell” and “cell part”, suggesting structural adjustments during developmental progression. In terms of molecular function, significant enrichment was observed in genes related to “binding” and “catalytic activity”, implying their central roles in senescence regulation. Additionally, genes associated with cell death, hormone response, and signal transduction were significantly enriched, indicating that these processes play key roles in regulating leaf growth, stress adaptation, and senescence mechanisms.

### 2.2. Screening for NAC Genes Associated with Leaf Senescence Among DEGs

Through comparative analysis of a full-length transcriptome aligned with the identified sequences with known *A. thaliana* NAC genes, we identified 57 *C. japonicum* NAC genes potentially involved in the regulation of leaf senescence (Table S6). Subsequently, these 57 NAC candidates were compared with the differentially expressed genes (DEGs) identified between the ULe and FLe stages. This comparison revealed nine NAC genes that were differentially expressed during senescence and are likely to participate in processes such as cell cycle regulation, cell differentiation, and responses to biotic and abiotic stresses (Table S7).

**Figure 2:**
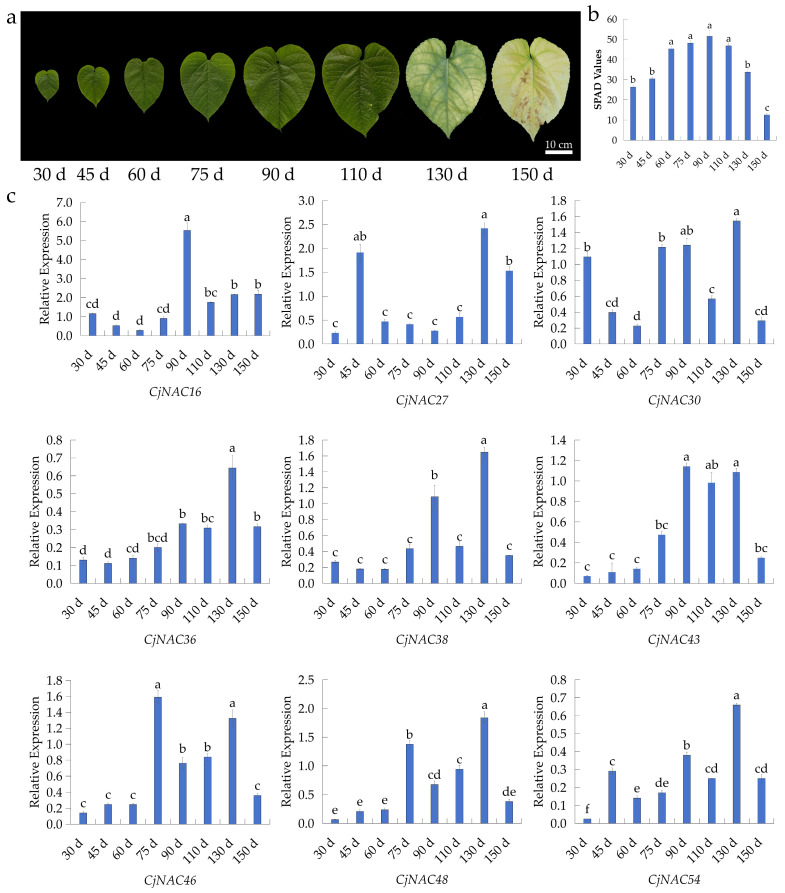
Temporal expression patterns of senescence-associated NAC genes in *C. japonicum*. (**a**) Phenotypic progression of leaf senescence from 30 to 150 days. (**b**) Dynamic changes in leaf SPAD values from 30 to 150 days. (**c**) Relative expression levels of nine selected NAC genes across six developmental stages (30, 45, 60, 75, 90, 110, 130, and 150 days) as determined by qRT-PCR. Data are normalized to the internal reference gene *CjUBQ-1* and presented as mean ± SD (*n* = 3). Different lowercase letters indicate significant differences among time points for each gene (one-way ANOVA followed by Tukey’s HSD test, *p* < 0.05).

To further investigate their temporal and spatial expression patterns and identify key regulators of senescence, phenotypic observations, SPAD value measurements, and quantitative real-time PCR (qRT-PCR) were performed across multiple developmental stages (30–150 days) in *C. japonicum* (Figure 2). Phenotypic observations of *C. japonicum* leaves from 30 to 150 days revealed that leaf yellowing initiated at 90 days and progressed to complete yellowing by 150 days (Figure 2a). Chlorophyll content (SPAD values) increased from 30 to 90 days, followed by a decline thereafter (Figure 2b). No significant differences were observed between day 75 and day 110 (Figure 1c), suggesting that chlorophyll degradation was not yet pronounced at the early senescent stage. However, a marked decline was observed beyond 90 days (Figure 2b). The expression levels of nine NAC genes were analyzed by qRT-PCR (Figure 2c).

The expression of *CjNAC16* peaked at 90 days, showing a significantly higher level than at other observation time points (Figure 2c). *CjNAC27* expression increased markedly at 45, 130 and 150 days, exceeding that observed at other stages (Figure 2c). *CjNAC30* exhibited fluctuating expression levels, with significantly higher expression at 30, 75, 90, and 130 days compared with other time points (Figure 2c). *CjNAC36* showed expression troughs at 30, 45, 60 and 75 days, which were significantly lower than those at other time periods (Figure 2c). *CjNAC38* displayed pronounced expression peaks at 90 and 130 days, significantly higher than at other stages (Figure 2c). The expression of *CjNAC43* increased significantly during the senescence stage (75–130 days), reaching higher levels than in earlier growth stages (Figure 2c). *CjNAC46* and *CjNAC48* exhibited a continuous upward trend in expression from 75 to 130 days, significantly higher than at other time points (Figure 2c). The expression pattern of *CjNAC54* was similar to that of *CjNAC43*, also showing a marked increase during the senescence stage (Figure 2c). The results showed that *CjNAC43* and *CjNAC54* exhibited markedly higher expression during the critical senescence phase (90–130 days), suggesting their important roles in the regulation of leaf aging. Based on their significant and sustained upregulation during natural senescence-a key criterion for identifying central regulators-we selected *CjNAC43* and *CjNAC54* for in-depth functional characterization.

Consequently, these two NAC genes were selected for subsequent functional analyses. Phylogenetic analysis using protein sequences of *A. thaliana* senescence-associated NAC genes further revealed that CjNAC43 is most closely related to ANAC002/ATAF1, whereas CjNAC54 shows the highest similarity to ANAC097, ANAC084, ANAC104.

### 2.3. Over-Expression of CjNAC43 and CjNAC54 Accelerates Leaf Senescence in Arabidopsis

**Figure 3:**
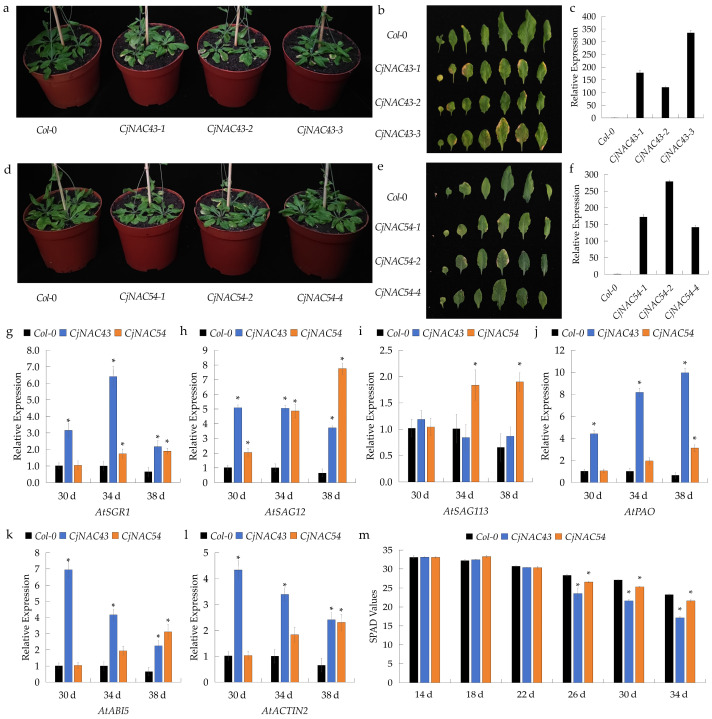
Overexpression of *CjNAC43* and *CjNAC54* accelerates leaf senescence in *A. thaliana*. (**a**,**d**) Whole-plant phenotype of *Col*-0, *CjNAC43*-overexpression (OE) lines, and *CjNAC54*-OE lines at 34 days after sowing. (**b**,**e**) Representative leaf images from the plants shown in (**a**,**d**) at 34 days. (**c**,**f**) Relative expression levels of *CjNAC43* (**c**) and *CjNAC54* (**f**) in *Col*-0 and homozygous T_3_ overexpression lines as determined by qRT-PCR. Data are mean ± SD (*n* = 3). (**g**–**l**) Relative expression levels of senescence-associated genes in *Col*-0 and OE lines at 30, 34, and 38 days: (**g**) *AtSGR1*, (**h**) *AtSAG12*, (**i**) *AtSAG113*, (**j**) *AtPAO*, (**k**) *AtABI5*, (**l**) *AtACTIN2*. Data are mean ± SD (*n* = 3). *, *p* < 0.05; vs. *Col*-0 at the same time point. (**m**) Temporal changes in leaf SPAD values of *Col*-0 and OE lines from 22 to 38 days. Data are mean ± SD (*n* ≥ 5). *, *p* < 0.05; vs. *Col*-0 at the same time point.

To investigate the biological functions of *CjNAC43* and *CjNAC54*, we successfully generated three homozygous T_3_ *A. thaliana* lines overexpressing each gene. Quantitative real-time PCR (qRT-PCR) analysis confirmed that all positive lines exhibited markedly elevated expression compared to the *Col*-0 plants (Figure 3c,f). Among them, lines with the highest transcript abundance (*CjNAC43-3* and *CjNAC54-2*) were selected for subsequent functional analysis (Figure 3c,f).

At 34 days after sowing, both *CjNAC43* and *CjNAC54* overexpression lines displayed visibly accelerated leaf senescence relative to *Col*-0 plants, with varying degrees of yellowing and chlorosis (Figure 3a,b,d,e). To further characterize the senescence phenotype at the molecular level, the expression patterns of several *A. thaliana* senescence-associated genes (SAGs) (*AtSGR1*, *AtSAG12*, and *AtSAG113*) as well as *AtPAO*, *AtABI5*, and *AtACTIN2* were analyzed by qRT-PCR at different developmental stages (Figure 3g–l).

*AtSGR1* and *AtSAG12* expression was significantly upregulated in both *CjNAC43*- and *CjNAC54*-overexpressing lines compared to *Col-0* between 30 and 38 days (Figure 3g,h). The expression of *AtSAG113* remained unchanged in *CjNAC43* overexpressors but was significantly higher in *CjNAC54*-overexpressing lines at 34 and 38 days (Figure 3i). Similarly, *AtPAO* expression increased progressively with advancing senescence in *CjNAC43* but was only significantly higher in *CjNAC54* plants than in *Col*-0 only at 38 days (Figure 3j). Interestingly, *AtABI5* expression was elevated, while *AtACTIN2* expression decreased; however, both showed significant differences from *Col*-0 at 38 days (Figure 3k,l).

In addition to molecular evidence, physiological measurements of chlorophyll content (SPAD values) further supported these observations. From day 26 onward, SPAD values of overexpression lines were significantly lower than those of *Col*-0 plants, with a more pronounced decline observed in *CjNAC43* plants (Figure 3m), consistent with their more severe senescence phenotype (Figure 3a,b,d,e).

### 2.4. CjNAC43 and CjNAC54 Mediates Leaf Senescence Induced by ABA Treatment and Darkness

To further elucidate the roles of *CjNAC43* and *CjNAC54* in abiotic stress-induced leaf senescence, we subjected their overexpression lines to abscisic acid (ABA) and darkness treatments.

**Figure 4:**
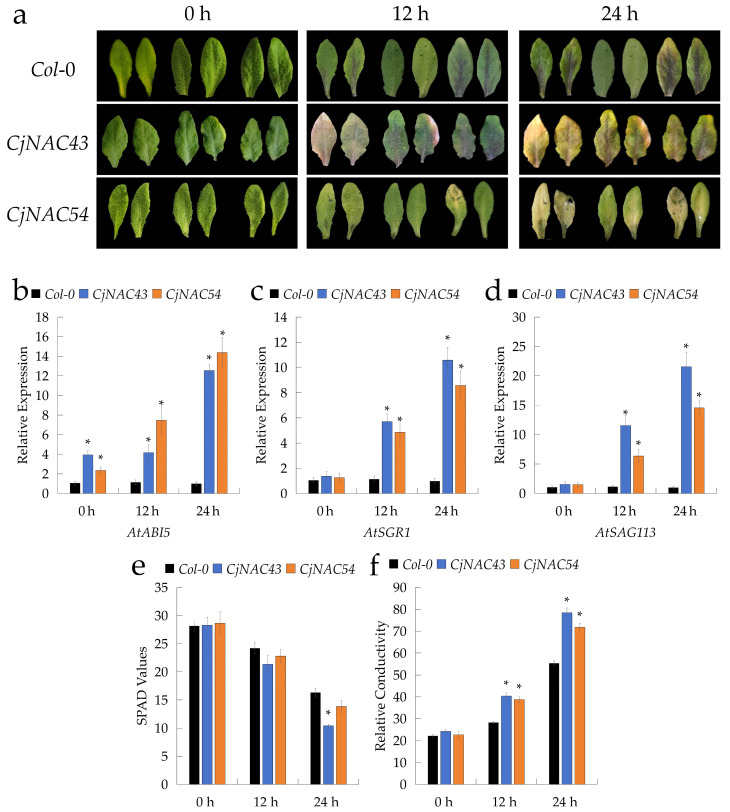
*CjNAC43* and *CjNAC54* mediate leaf senescence induced by abscisic acid (ABA) treatment. (**a**) Phenotypic changes of detached leaves from *Col*-0, *CjNAC43*-OE, and *CjNAC54*-OE plants at 0, 12, and 24 h after treatment with 10 μM ABA. (**b**–**d**) Relative expression levels of ABA- and senescence-responsive genes: (**b**) *AtABI5*, (**c**) *AtSGR1*, (**d**) *AtSAG113* at 0, 12, and 24 h post-ABA treatment. Data are mean ± SD (*n* = 3). *, *p* < 0.05 vs. *Col-0* at the same time point. Different lowercase letters indicate significant differences among time points within each genotype. (**e**) Changes in leaf SPAD values following ABA treatment. Data are mean ± SD (*n* ≥ 5). *, *p* < 0.05 vs. *Col-0* at the same time point. (**f**) Changes in leaf relative ion conductivity (an indicator of membrane integrity) following ABA treatment. Data are mean ± SD (*n* = 3). *, *p* < 0.05 vs. *Col-0* at the same time point.

Following foliar application of 10 μM ABA, the fifth and sixth leaves of four-week-old overexpressing *A. thaliana* plants began to exhibit visible yellowing within 12 h (Figure 4a). At the molecular level, quantitative analyses revealed that the expression levels of *AtABI5*, *AtSGR1*, and *AtSAG113* progressively increased in both overexpression lines at 0, 12, and 24 h after ABA treatment (Figure 4b–d). Concurrently, relative ion conductivity also increased, while SPAD values decreased, with *CjNAC43*-overexpressing lines showing a more pronounced reduction in chlorophyll content than *CjNAC54*-overexpressing lines (Figure 4e,f). These results indicate that *CjNAC43* and *CjNAC54* enhance ABA sensitivity, thereby accelerating the onset of senescence.

**Figure 5:**
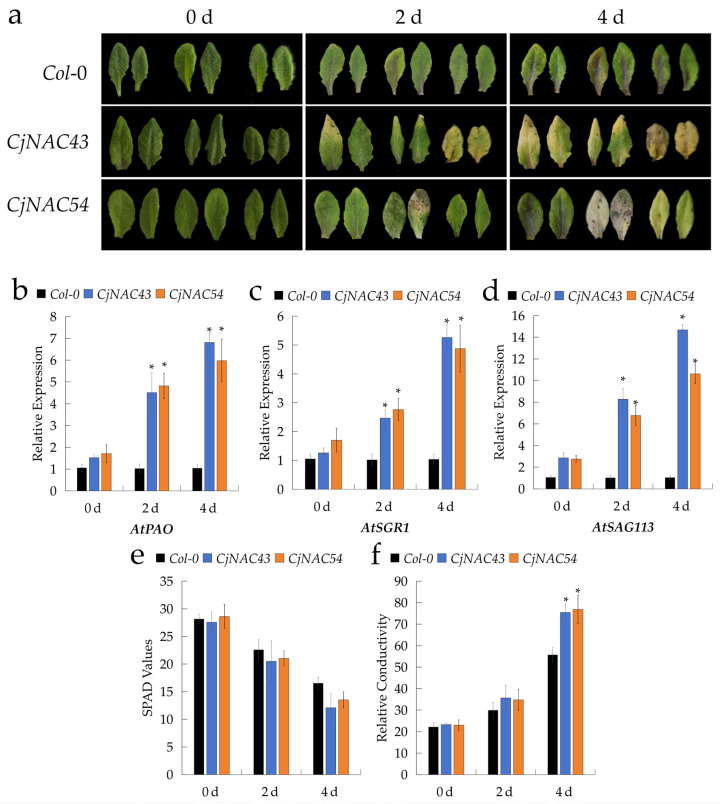
*CjNAC43* and *CjNAC54* mediate leaf senescence induced by darkness. (**a**) Phenotypic changes of detached leaves from *Col*-0, *CjNAC43*-OE, and *CjNAC54*-OE plants at 0, 2, and 4 days of dark treatment. (**b**–**d**) Relative expression levels of senescence-related genes under dark treatment: (**b**) *AtPAO*, (**c**) *AtSGR1*, (**d**) *AtSAG113*. Data are mean ± SD (*n* = 3). *, *p* < 0.05 vs. *Col-0* at the same time point. Different lowercase letters indicate significant differences among time points within each genotype. (**e**) Changes in leaf SPAD values under dark treatment. Data are mean ± SD (*n* ≥ 5). *, *p* < 0.05 vs. *Col-0* at the same time point. (**f**) Changes in leaf relative ion conductivity under dark treatment. Data are mean ± SD (*n* = 3). *, *p* < 0.05 vs. *Col-0* at the same time point.

Similarly, under darkness treatment, the fifth and sixth leaves of four-week-old overexpressing *A. thaliana* plants began to yellow within 12 h (Figure 5a), closely resembling the phenotypes observed under ABA treatment. At 0, 12, and 24 h of dark exposure, the transcript levels of *AtPAO*, *AtSGR1*, and *AtSAG113*, as well as relative ion conductivity, exhibited a consistent upward trend (Figure 5b–d,f), whereas SPAD values declined correspondingly (Figure 5e). These physiological and molecular responses were in agreement with the visible senescence phenotype, suggesting that *CjNAC43* and *CjNAC54* act as positive regulators of leaf senescence under both ABA and dark stress conditions.

### 2.5. Suppression of CjNAC43 and CjNAC54 Expression Results in Delayed Leaf Senescence

To further investigate the functions of *CjNAC43* and *CjNAC54* in *C. japonicum*, we first established a virus-induced gene silencing (VIGS) system using the Tobacco Rattle Virus (TRV) vector. The phytoene desaturase (PDS) gene, a key gene involved in photosynthetic pigment biosynthesis, was selected as a visual marker. Using *Agrobacterium*-mediated transformation, efficient infection of *C. japonicum* seedlings was achieved. Leaf albinism began to appear eight days after infection and became more pronounced by day 14 (Figure S3a), confirming the successful establishment of a functional VIGS system in *C. japonicum*. Quantitative real-time PCR analysis further verified that the relative expression level of PDS in *pTRV2-CjPDS* plants decreased by approximately 81% compared to the negative control (pTRV1+pTRV2), demonstrating effective gene silencing (Figure S3b).

**Figure 6:**
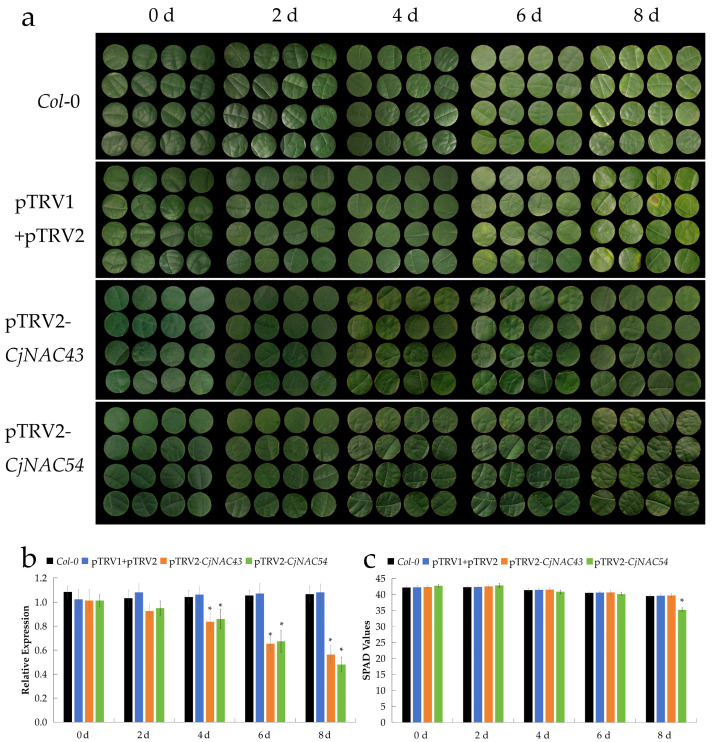
Suppression of *CjNAC43* and *CjNAC54* expression delays leaf senescence in *C. japonicum* via VIGS. (**a**) Phenotypic comparison of leaves from *Col*-0, empty vector control (pTRV1+pTRV2), *CjNAC43*-silenced (*pTRV2-CjNAC43*), and *CjNAC54*-silenced (*pTRV2-CjNAC54*) plants at 0, 2, 4, 6, and 8 days post-agroinfiltration. (**b**) Relative expression levels of *CjNAC43* and *CjNAC54* in the corresponding plants at different time points, as determined by qRT-PCR. Data are normalized to *CjUBQ-1* and presented as mean ± SD (*n* = 3). *, *p* < 0.05 vs. empty vector control at the same time point. (**c**) Comparison of leaf SPAD values among the different groups at 8 days post-infiltration. Data are mean ± SD (*n* ≥ 5). *, *p* < 0.05 vs. empty vector control.

Subsequently, recombinant silencing expression vectors targeting *CjNAC43* and *CjNAC54* were successfully constructed in *C. japonicum*, providing a reliable tool for functional validation of senescence-related NAC genes. Following *Agrobacterium*-mediated infection with the silencing constructs, leaves of both *CjNAC43*- and *CjNAC54*-silenced plants exhibited a visibly greener phenotype compared with the empty vector controls by day 8. Correspondingly, their relative expression levels were significantly reduced from day 4 onward, indicating that suppression of *CjNAC43* and *CjNAC54* expression delayed leaf senescence. Notably, the expression levels of both silenced genes remained low over time, with *CjNAC54*-silenced leaves showing a more pronounced decrease in transcript abundance on day 8 compared to *CjNAC43*-silenced leaves (Figure 6a,b). Consistently, SPAD values were significantly higher in the silenced plants on day 8 post-infiltration (Figure 6c).

## 3. Discussion

Leaf senescence, a programmed developmental process crucial for plant fitness and resource allocation, is orchestrated by a complex regulatory network in which NAC transcription factors play pivotal roles [13,22]. In this study, we demonstrate that *CjNAC43* and *CjNAC54* function as positive regulators of leaf senescence in the medicinal and ornamental woody shrub *C. japonicum*. Our conclusions are supported by a combination of transcriptomic profiling, phylogenetic analysis, and robust functional evidence from both heterologous overexpression in *A. thaliana* and VIGS-mediated gene silencing in the native host.

Our initial transcriptomic analysis of mature (ULe) and early senescent (FLe) leaves identified a suite of differentially expressed genes, among which nine were NAC family members. The distinct upregulation of *CjNAC43* and *CjNAC54* during the critical senescence window (90–130 days) marked them as prime candidates for regulators of this process (Figure 2). Phylogenetic reconstruction further contextualized their potential functions, revealing that CjNAC43 shares the closest homology with ATAF1 (ANAC002), while CjNAC54 clusters with ANAC097, ANAC084, ANAC104. Notably, ATAF1 is an established positive regulator of senescence in *A. thaliana* [46]. This phylogenetic conservation suggests that the regulatory mechanisms governing senescence are, to some extent, evolutionarily conserved between herbaceous model plants and woody species. ATAF1 is known to integrate stress and ABA signaling pathways to promote senescence [44,46], and ANAC097 has been implicated in reactive oxygen species (ROS)-mediated cell death and leaf aging. The conserved senescence-promoting role of these NAC orthologs in the distantly related woody species *C. japonicum* highlights the evolutionary significance of this transcriptional regulatory module in managing leaf lifespan.

The most compelling evidence for the functions of *CjNAC43* and *CjNAC54* comes from our gain-of-function and loss-of-function studies. Heterologous overexpression of either gene in *A. thaliana* consistently induced precocious leaf senescence, characterized by accelerated chlorophyll degradation and the significant upregulation of classic senescence-associated genes (*SAG12*, *SAG113*) and chlorophyll catabolic genes (*PAO*) (Figure 3). This phenotype is reminiscent of that observed upon overexpression of their Arabidopsis homologs, reinforcing the idea of functional conservation. Crucially, the establishment of a VIGS system in *C. japonicum* enabled the direct functional interrogation of these genes in their native context. Silencing of *CjNAC43* or *CjNAC54* resulted in a visibly delayed senescence phenotype, accompanied by higher retention of chlorophyll (Figure 6). This finding not only confirms their pro-senescence function but also indicates that these transcription factors are physiologically relevant regulators of the natural senescence program in *C. japonicum*, beyond merely inducing senescence upon artificial overexpression. Our reciprocal genetic evidence from both heterologous overexpression and VIGS-mediated silencing strongly supports the conclusion that *CjNAC43* and *CjNAC54* are key regulators of leaf senescence in *C. japonicum*. This reciprocal evidence from two independent experimental systems provides a solid chain of causation, unequivocally establishing *CjNAC43* and *CjNAC54* as key drivers of leaf senescence in *C. japonicum*.

To dissect the mechanistic basis of their action, we investigated how *CjNAC43* and *CjNAC54* mediate senescence triggered by hormonal and environmental cues. We found that both genes are integral to the senescence response induced by ABA and darkness. The overexpression lines exhibited heightened sensitivity to both treatments, manifesting as more rapid leaf yellowing, a sharper decline in SPAD values, increased ion leakage, and hyper-induction of key marker genes such as *ABI5*, *SGR1*, and *SAG113* (Figure 4 and Figure 5). This positions *CjNAC43* and *CjNAC54* as critical nodes downstream of ABA and dark signaling pathways, amplifying these signals to execute the senescence program. Specifically, the upregulation of *ABI5* (a core component of ABA signaling) and *SGR1* (a key chlorophyll degradation enzyme) directly links *CjNAC43*/*54* activity to both the hormonal signaling cascade and the executive machinery of chlorophyll breakdown. This role is consistent with the well-documented function of NAC TFs as central integrators of diverse internal and external senescence-promoting signals [14,46].

Interestingly, we observed subtle yet notable differences between the two genes. While both positively regulate senescence, their modes of action appear non-redundant. For instance, *AtSAG113* expression was more strongly affected by *CjNAC54* overexpression, and the chlorophyll loss under ABA stress was more pronounced in *CjNAC43* lines. This synergistic yet distinct involvement is further supported by their responses to different stresses: *CjNAC43* overexpression led to a more rapid chlorophyll degradation under ABA treatment, whereas both genes were equally potent in accelerating dark-induced senescence. These observations suggest potential functional diversification, where *CjNAC43* and *CjNAC54* may regulate partially overlapping but distinct subsets of downstream target genes. It is plausible that *CjNAC43* is more potent in transducing ABA-mediated senescence signals, while *CjNAC54* might be more specialized in regulating a different branch of the senescence network, such as one involving specific SAGs or ROS homeostasis. This specialization could enable the plant to fine-tune the senescence response based on the specific environmental cue (e.g., drought vs. shading) or internal hormonal balance. This functional divergence, despite their concurrent upregulation during senescence, could allow for fine-tuned regulation of the complex senescence process in response to multiple inputs, an intriguing aspect that warrants further investigation.

This research provides one of the first functional characterizations of senescence-associated NAC TFs in the genus *Clerodendrum*. While numerous NAC genes have been studied in model herbaceous plants [12,13,47], and their roles in stress-induced senescence have been elucidated in crops [34,35,36], their functions in woody species, particularly those of high ornamental value, remain relatively unexplored [37,38]. Our work bridges this knowledge gap and extends the paradigm of NAC-mediated senescence regulation to a novel, economically important plant species. Furthermore, the successful implementation of the VIGS system in *C. japonicum* establishes a valuable reverse-genetics platform for future functional genomics studies in this and related species. This technical advancement is significant, as genetic studies in non-model woody plants are often hindered by long life cycles and difficulties in stable transformation.

In conclusion, our data establish a model in which *CjNAC43* and *CjNAC54* act as master positive regulators of leaf senescence in *C. japonicum*. They function by integrating the ABA hormone pathway and the environmental dark signal, thereby activating the expression of executor genes responsible for chlorophyll degradation and cellular dismantling. Beyond advancing our fundamental understanding of leaf senescence in woody plants, these findings open up potential avenues for biotechnological applications. Future research should focus on identifying the direct genomic targets of *CjNAC43* and *CjNAC54* through techniques like ChIP-seq, which will elucidate the precise architecture of the downstream regulatory network. Moreover, exploring the potential of these genes as targets for genetic or biotechnological strategies to delay senescence-for instance, by using tissue-specific or stress-inducible promoters to drive RNAi constructs-could enhance the ornamental value and stress resilience of *C. japonicum* and other economically important woody plants.

## 4. Materials and Methods

### 4.1. Plant Meterial and Transcriptome Sequencing

Three-year-old *C. japonicum* were cultivated in the nursery of the Guangdong Academy of Forestry Sciences (23°11′59″ N, 113°22′11″ E). Total RNA was extracted from mature (75 days after leaf emergence, ULe) and early senescent (110 days after leaf emergence, FLe) leaves of *C. japonicum*. The selection of these two critical time points was based on comprehensive preliminary phenotyping and physiological monitoring (as shown in Figure 2a,b): The 75-day (ULe) time point represents the stage of full leaf maturity and maximum photosynthetic competence, immediately prior to the onset of any visible senescence symptoms (see Figure 2a, 75 d). This stage serves as the optimal baseline for pre-senescence transcriptomic profiling. The 110-day (FLe) time point was identified as the early senescence phase, characterized by the initial visual signs of chlorophyll degradation (leaf yellowing) and concurrent floral bud development (see Figure 1b and Figure 2a, 110 d). Sampling at this stage allows for the capture of transcriptional reprogramming associated with the initiation of the senescence process. (A schematic diagram illustrating the plant architecture, standardized leaf sampling positions, and the rationale for the selected time points is provided in Supplementary Figure S1.)

Total RNA was extracted from mature (75 days, ULe) and early senescent (110 days, FLe) leaves of *C. japonicum* using a Plant RNA Kit (Omega Biotech, Norcross, GA, USA), with three biological replicates for each developmental stage. RNA sequencing (RNA-Seq) was conducted by Gene Denovo Co. (Guangzhou, China) using the Illumina HiSeq 2000 platform with a paired-end sequencing strategy.

Raw reads were subjected to quality control using fastp [48] to remove low-quality sequences and adapter contamination. A summary of the RNA-Seq data quality metrics is provided in Supplementary Table S1. The resulting clean reads were aligned and quantified using RSEM [49] with the *C. japonicum* full-length transcriptome (National Genomics Data Center, BioProject: PRJCA019175) as a reference. Gene expression levels were calculated for each sample based on fragments per kilobase of transcript per million mapped reads (FPKM).

### 4.2. Differential Gene Analysis

Based on third-generation full-length transcriptome sequencing technology (PacBio) [50], single-molecule real-time sequencing (SMRT) was used to obtain high-quality, full-length transcripts of *C. japonicum*.

Differentially expressed genes (DEGs) between the ULe and FLe stages were identified using DESeq2 [51], applying a screening threshold of false discovery rate (FDR) < 0.05 and |log_2_FC| > 1. Functional annotation of the DEGs was performed by BLASTX (Version 2.14.0) alignment against the NCBI non-redundant protein (Nr) database (e-value < 1 × 10^−5^). Gene Ontology (GO) terms were assigned using Blast2GO v5.0 based on the Nr annotation results. Kyoto Encyclopedia of Genes and Genomes (KEGG) pathway annotations were obtained using the KEGG Automatic Annotation Server (KAAS). For enrichment analysis, all annotated genes were used as the background reference. GO enrichment analysis was conducted using the GOseq R package (Version 2.11.0) based on the Wallenius non-central hypergeometric distribution, which accounts for gene length bias [52]. KEGG pathway enrichment analysis was performed using KOBAS 3.0 with a hypergeometric test [53]. Terms and pathways with a corrected *p*-value (FDR) < 0.05 were considered significantly enriched.

### 4.3. Identification of NAC Family

All NAC protein sequences were extracted using a Perl script [54] and aligned against the Plant Transcription Factor Database (PlantTFDB [24], https://planttfdb.gao-lab.org/, accessed on 1 May 2025), using HMMscan to identify members of the *C. japonicum* NAC family.

### 4.4. Relative Expression Measurement

Varieties of *C. japonicum* were selected to investigate differential gene expression during leaf senescence (30–150 days). Total RNA was extracted using the Total RNA Extraction Kit (HYY0420-50; Beijing Huayueyang Co., Ltd., Beijing, China, http://www.huayueyang.com.cn/), and cDNA was synthesized following the manufacturer’s instructions for the StarScript II One-Step RT-PCR Kit (K215-04; Shanghai Kanglang Biotechnology Co., Ltd., Shanghai, China, http://www.klbscience.com/). qRT-PCR primers were designed using Primer 6.0 software [55] and synthesized by Shanghai Sangon Biological Co., Ltd. (Shanghai, China). *CjUBQ-1* served as the internal reference gene for *C. japonicum*, while *AtUBQ* was used as the internal control for *A. thaliana*. Each treatment included three biological replicates, and relative gene expression levels were calculated using the 2^−ΔΔCT^ method.

### 4.5. Measurement of Physiological Parameters

Chlorophyll content was estimated non-destructively using a SPAD-502 chlorophyll meter (Konica Minolta, Tokyo, Japan). Measurements were taken at the midpoint between the leaf margin and the midvein, avoiding major veins. Three readings were taken per leaf and averaged to represent a single biological replicate.

Relative ion conductivity, as an indicator of membrane integrity and cell senescence, was determined according to an established electrolyte leakage protocol [56]. Briefly, five leaf discs (6 mm in diameter) were excised using a cork borer, rinsed three times with deionized water to remove surface electrolytes, and then floated in 10 mL of deionized water in a sealed tube. The samples were incubated at room temperature with gentle shaking for 3 h, after which the initial conductivity (C1) of the bathing solution was measured using a conductivity meter (DDSJ-308A, INESA Scientific Instrument Co., Ltd., Shanghai, China). The samples were then boiled at 100 °C for 15 min to release all electrolytes, cooled to room temperature, and the final conductivity (C2) was measured. Relative ion conductivity was calculated as (C1/C2) × 100%. Three independent biological replicates were performed for each measurement [56,57].

### 4.6. Phylogenetic Analysis

A phylogenetic tree was constructed using RAxML with the neighbor-joining method and 1000 bootstrap replicates [58], based on protein sequences of NAC family previously reported to be involved in *A. thaliana*. All 138 NAC reference protein sequences were obtained from PlantTFDB [24] (https://planttfdb.gao-lab.org/ (accessed on 1 May 2025)).

### 4.7. Construction of Overexpression Vector

Specific primers for the key NAC target gene of *C. japonicum* were designed using Primer Premier 6.0 software [55] and synthesized by Shanghai Sangon Biotechnology Co., Ltd. Using the cDNA of *C. japonicum* as a template, the target fragment was amplified by PCR with a high-fidelity enzyme (PrimerStar Max Premix, Takara Bio Inc. Kusatsu, Japan). The overexpression vector pGreen-C1 was utilized, and the amplified fragment was inserted via homologous recombination following the instructions of the ClonExpress II One-Step Cloning Kit produced by Nanjing Vazyme Biotech Co., Ltd. (Nanjing, China).

### 4.8. Generation of Transgenic Plants

The recombinant plasmid was introduced into *Escherichia coli* DH5α competent cells and plated on LB medium containing the appropriate antibiotic (Primer sequences are listed in Supplementary Table S8). Positive colonies were identified by colony PCR and restriction enzyme digestion, and plasmids from verified clones were extracted. The confirmed recombinant plasmids were subsequently transformed into *Agrobacterium tumefaciens* GV3101 (pSoup).

Transgenic *A. thaliana* plants were generated using the floral-dip method. T_0_ seeds were collected, sown in nutrient soil, and sprayed with 0.1% Basta during the seedling stage to select T_1_ plants carrying the bar resistance gene. Surviving seedlings were verified by PCR, and positive T_1_ individuals were self-pollinated to obtain T_2_ seeds. The T_2_ generation was further screened for herbicide resistance and molecular confirmation. Homozygous T_3_ lines were identified and used for subsequent functional analyses.

### 4.9. Phenotypic and Functional Analysis of Overexpressing A. thaliana Lines

To elucidate the function of the target gene, homozygous *A. thaliana* lines overexpressing the gene were phenotypically characterized, with wild-type (*Col-0*) plants serving as controls.

The expression patterns of senescence-associated genes were examined using quantitative real-time PCR (qRT-PCR) (Primer sequences are listed in Supplementary Table S9). Leaf samples were collected at 30, 34, and 38 days after sowing, with three biological replicates per time point. The expression levels of endogenous senescence marker genes (*AtSGR1*, *AtSAG12*, *AtSAG113*, *AtPAO*, *AtABI5* and *AtACTIN2*) were quantified, using *Col-0* as the reference.

Chlorophyll content was measured using a SPAD-502 chlorophyll meter (Konica Minolta, Tokyo, Japan), with three biological replicates for each time point. Relative ion leakage was determined to assess membrane integrity. Briefly, leaf discs were cut and immersed in distilled water, sealed, and shaken at room temperature for 12 h to obtain the initial conductivity (S_1_). Samples were then boiled for 15 min, cooled to room temperature, and measured again to obtain the final conductivity (S_2_). Relative ion leakage was calculated as (S_1_/S_2_) × 100%. All assays for measuring relative ion leakage were performed with three biological replicates.

### 4.10. Abiotic Stress Treatment

For the abscisic acid (ABA) treatment, the fifth or sixth fully expanded leaves from four-week-old transgenic *A. thaliana* plants were selected. Leaves from the control group were sprayed with distilled water, whereas those in the experimental group were sprayed with 10 μM ABA solution. The treated leaves were placed on filter paper moistened with distilled water in Petri dishes, which were sealed with parafilm to maintain humidity, and incubated at 25 °C. Phenotypic changes were recorded at different time points. Leaf samples were collected at 0, 12, and 24 h after treatment, with wild-type (*Col-0*) plants serving as controls. The expression dynamics of senescence-related genes (*AtABI5*, *AtSGR1*, and *AtSAG113*) were analyzed by qRT-PCR. SPAD values and relative ion leakage were determined as described in Section 4.4. Three biological replicates were included for each time point.

For the dark-induced senescence assay, the fifth or sixth leaves from four-week-old *Col-0* and transgenic *A. thaliana* plants were detached and placed on filter paper pre-moistened with 2 mM MES buffer and 1/2 MS solution. Each Petri dish was tightly sealed with sterile aluminum foil to ensure darkness and incubated at 25 °C for 4 days. Samples were collected from *Col-0* and transgenic plants (*n* = 3) at 0, 12, and 24 h after dark treatment. The expression patterns of *AtPAO*, *AtSGR*, and *AtSAG113* were analyzed using qRT-PCR, and SPAD values and relative ion leakage were determined according to Section 4.4. Three biological replicates were included for each time point.

### 4.11. VIGS System Construction and Functional Verification

Based on the *A. thaliana* PDS gene sequence, specific VIGS primers were designed using Primer 6.0 [55] (Table S9), and a PDS gene fragment was cloned from *C. japonicum*. The fragment was ligated into the pTRV2 vector, followed by transformation into *Escherichia coli* DH5α. Positive clones were identified, extracted, and subsequently transformed into *Agrobacterium tumefaciens* GV3101 as described in Section 4.3. The resulting bacterial suspensions were mixed at equal volumes of pTRV1 and pTRV2 (empty vector control) and *pTRV2-CjPDS* (experimental group), respectively, and used to infect *C. japonicum* leaves for 4 h.

After infection, the plants were kept in darkness for 24 h, and then transferred to a growth chamber maintained at 24 °C under a 16 h light/8 h dark photoperiod. Phenotypic changes were monitored, and leaf samples were collected at appropriate time points. RNA extraction was performed as described in Section 4.2. Quantitative PCR analysis of PDS gene expression were conducted following the procedures in Section 4.4. The VIGS vector construction and infection procedures for the NAC genes followed the same method as for PDS (Primer sequences are listed in Supplementary Table S10). SPAD measurement, and ion permeability assays were conducted following the procedures in Section 4.4. Each treatment included three biological replicates.

### 4.12. Statistical Analysis

All quantitative data are presented as the mean ± standard deviation (SD) of at least three independent biological replicates. For comparisons between two groups (e.g., transgenic line vs. wild type at a single time point), statistical significance was assessed using a two-tailed Student’s *t*-test. For comparisons among multiple groups (e.g., gene expression across different time points, or multiple treatment groups), one-way analysis of variance (ANOVA) was first performed. When ANOVA indicated a significant overall effect (*p* < 0.05), Tukey’s honestly significant difference (HSD) post-hoc test was applied for pairwise comparisons between group means. Statistical analyses were performed using SPSS software (Version 26.0; IBM Corp., Armonk, NY, USA). Differences were considered statistically significant at *p* < 0.05. In figures, significant differences are denoted by asterisks (*) for *t*-test results or by different lowercase letters (a, b, c) for results of ANOVA followed by Tukey’s HSD test with a significance threshold of *p* < 0.05.

## 5. Conclusions

In this study, we integrated transcriptomic profiling, heterologous overexpression, and VIGS-based functional analyses to elucidate the roles of NAC transcription factors in the leaf senescence of *C. japonicum*. Among nine senescence-associated NAC candidates, *CjNAC43* and *CjNAC54* were identified as key regulators due to their marked upregulation during natural aging. Functional characterization demonstrated that overexpression of either gene in *A. thaliana* accelerated leaf senescence, promoted the expression of SAGs, and reduced chlorophyll content, whereas their silencing in *C. japonicum* delayed senescence. Furthermore, both genes enhanced ABA and dark sensitivity, indicating that they modulate stress-induced senescence through hormonal and environmental signaling pathways. Collectively, these findings establish *CjNAC43* and *CjNAC54* as positive regulators of leaf senescence in *C. japonicum* and provide a molecular basis for future breeding efforts aimed at optimizing senescence progression in medicinal and ornamental plants.

## Data Availability

The raw RNA-Seq data generated in this study have been deposited in the Genome Sequence Archive (GSA) at the China National Center for Bioinformation (CNCB) under BioProject accession number PRJCA019175. The data are accessible via the CNCB submission portal (https://ngdc.cncb.ac.cn/gsub/, accessed on 22 August 2023) under GSA accession number CRA012295.

## Supplementary Materials

The following supporting information can be downloaded at: https://www.mdpi.com/article/10.3390/ijms27010133/s1.

## Abbreviations

The following abbreviations are used in this manuscript:

ABA	Abscisic acid
ET	Ethylene
SGR	STAY-GREEN
PAO	PHEOPHORBIDE A OXYGENASE
SAG	senescence-associated genes
ACT	ACTIN
NAC	NAM/ATAF/CUC
TF	Transcription factors
VIGS	Virus-induced gene silencing
SPAD	Soil and Plant Analyzer Development
GC	Guanine-cytosine
FPKM	Fragments Per Kilobase of exon model per Million mapped fragments
ATP	Adenosine triphosphate
DEG	Differential expressed genes
KEGG	Kyoto Encyclopedia of Genes and Genomes
GO	Gene Ontology
PDS	Phytoene desaturase
