# Supplementary material for: The NAC Transcription Factors CjNAC43 and CjNAC54 Act as Positive Regulators of Leaf Senescence in Clerodendrum japonicum
Source: Int J Mol Sci. 2025 Dec 22;27(1):133. doi: 10.3390/ijms27010133 (PMC12785693; doi:10.3390/ijms27010133)
Supplement: Supplementary file 1 [file ijms-27-00133-s001.zip › Figure S1. Schematic diagram of leaf sampling and phenotypic progression in Clerodendrum japonicum..pdf]

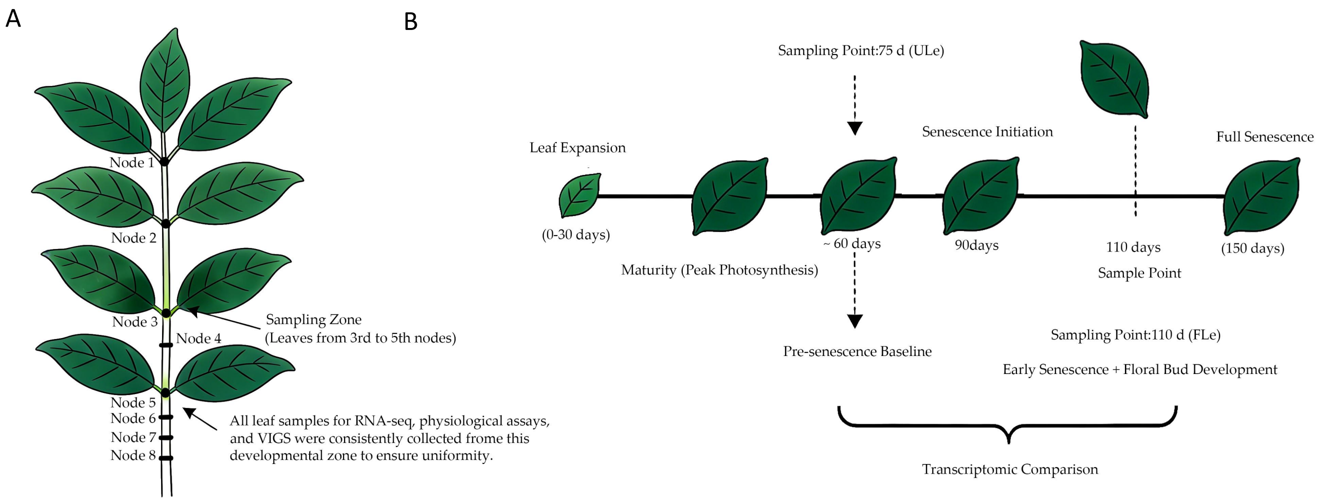

Supplementary Figure S1. Schematic representation of plant growth, leaf sampling strategy, and senescence progression in Clerodendrum japonicum. (A) Diagram of a mature C. japonicum plant indicating the nodal positions and the standardized sampling zone (leaves at the 3rd to 5th nodes). (B) Simplified timeline of leaf development and senescence, showing key physiological stages and the two sampling time points (75 and 110 days after leaf emergence) used for transcriptomic analysis.
