# Supplementary material for: The NAC Transcription Factors CjNAC43 and CjNAC54 Act as Positive Regulators of Leaf Senescence in Clerodendrum japonicum
Source: Int J Mol Sci. 2025 Dec 22;27(1):133. doi: 10.3390/ijms27010133 (PMC12785693; doi:10.3390/ijms27010133)
Supplement: Supplementary file 1 [file ijms-27-00133-s001.zip › Figure S2. Phylogenetic analysis of CjNAC43 and CjNAC54 with NAC Proteins in Arabidopsis thaliana.pdf]

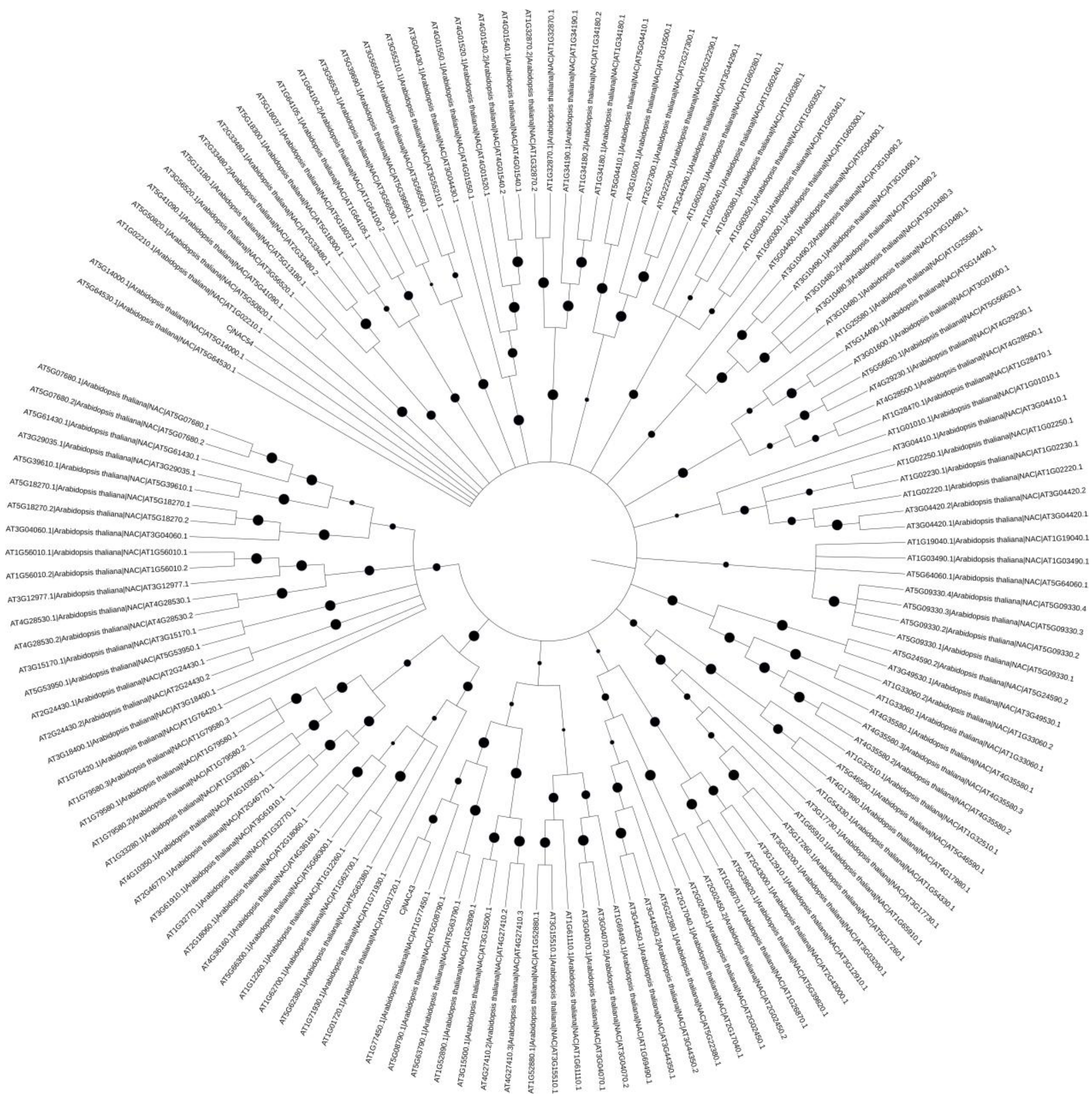

Figure S2. Phylogenetic analysis of *CjNAC43* and *CjNAC54* with NAC Proteins in *Arabidopsis thaliana*. Phylogenetic tree was constructed using RAxML with the neighbor-joining method and 1,000 bootstrap replicates, based on protein sequences of NAC family previously reported to be involved in A. thaliana. All 138 NAC reference protein sequences were obtained from PlantTFDB (https://planttfdb.gao-lab.org/).
