# Supplementary material for: The NAC Transcription Factors CjNAC43 and CjNAC54 Act as Positive Regulators of Leaf Senescence in Clerodendrum japonicum
Source: Int J Mol Sci. 2025 Dec 22;27(1):133. doi: 10.3390/ijms27010133 (PMC12785693; doi:10.3390/ijms27010133)
Supplement: Supplementary file 1 [file ijms-27-00133-s001.zip › Figure S3. Validation of the Virus-Induced Gene Silencing (VIGS) system in Clerodendrum japonicum using.pdf]

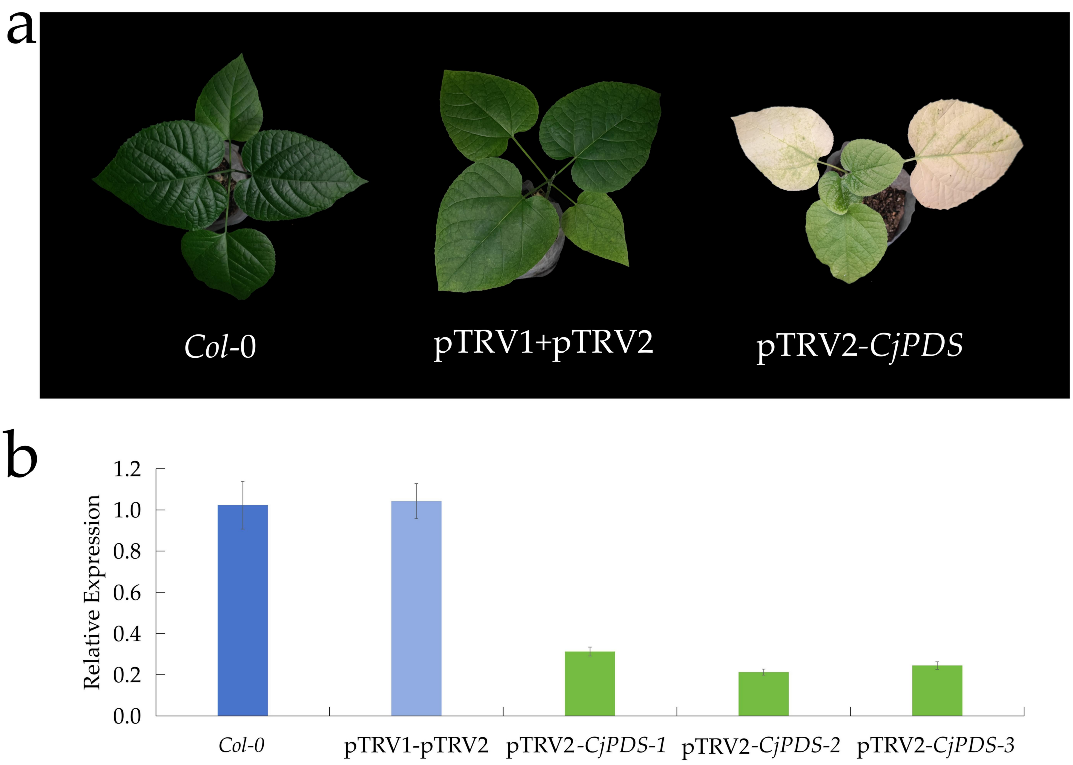

Figure S3. Validation of the Virus-Induced Gene Silencing (VIGS) system in *Clerodendrum japonicum* using the Phytone Desaturase (*PDS*) gene. (a) Leaf phenotypes showing albinism (photobleaching) in *C. japonicum* seedlings infected with the *pTRV2-CjPDS* construct at 8 and 14 days post-infiltration (dpi), compared to the empty vector control (pTRV1+pTRV2). The albino phenotype indicates successful silencing of the PDS gene. (b) Relative expression level of *CjPDS* in leaves infected with *pTRV2-CjPDS* compared to the empty vector control, as determined by qRT-PCR at 14 dpi. Data are mean ± SD (n = 3). **, $p < 0.01$.
