# Supplementary material for: The NAC Transcription Factors CjNAC43 and CjNAC54 Act as Positive Regulators of Leaf Senescence in Clerodendrum japonicum
Source: Int J Mol Sci. 2025 Dec 22;27(1):133. doi: 10.3390/ijms27010133 (PMC12785693; doi:10.3390/ijms27010133)
Supplement: Supplementary file 1 [file ijms-27-00133-s001.zip › Table S1 Summary of RNA-Seq data quality control.pdf]

Table S1. Summary of RNA-Seq data quality control.

Sample	RawDatas	CleanData(%)	Adapter(%)	LowQuality(%)	polyA(%)	N(%)
Cj-FLe-1	45409428	45327492 (99.82%)	25294 (0.06%)	56638 (0.12%)	0 (0.00%)	4 (0.00%)
Cj-FLe-2	36822468	36751634 (99.81%)	23102 (0.06%)	47730 (0.13%)	0 (0.00%)	2 (0.00%)
Cj-FLe-3	49847820	49775150 (99.85%)	18946 (0.04%)	53722 (0.11%)	0 (0.00%)	2 (0.00%)
Cj-ULe-1	45373166	45321320 (99.89%)	14824 (0.03%)	37012 (0.08%)	0 (0.00%)	10 (0.00%)
Cj-ULe-2	42537080	42476800 (99.86%)	16000 (0.04%)	44272 (0.10%)	0 (0.00%)	8 (0.00%)
Cj-ULe-3	56710880	56630072 (99.86%)	18624 (0.03%)	62178 (0.11%)	0 (0.00%)	6 (0.00%)
