# Supplementary material for: The NAC Transcription Factors CjNAC43 and CjNAC54 Act as Positive Regulators of Leaf Senescence in Clerodendrum japonicum
Source: Int J Mol Sci. 2025 Dec 22;27(1):133. doi: 10.3390/ijms27010133 (PMC12785693; doi:10.3390/ijms27010133)
Supplement: Supplementary file 1 [file ijms-27-00133-s001.zip › Table S2 Base composition and GC content of RNA-Seq data.pdf]

Table S2. Base composition and GC content of RNA-Seq data.

Sample	RawData(bp)	BF_Q20(%)	BF_Q30(%)	BF_N(%)	BF_GC(%)	CleanData(bp)	AF_Q20(%)	AF_Q30(%)	AF_N(%)	AF_GC(%)
Cj-FLe-1	6811414200	6664482230 (97.84%)	6392952412 (93.86%)	88104 (0.00%)	3260487385 (47.87%)	6729101600	6591783543 (97.96%)	6326647805 (94.02%)	86993 (0.00%)	3215069057 (47.78%)
Cj-FLe-2	5523370200	5410268869 (97.95%)	5194257045 (94.04%)	71182 (0.00%)	2636433622 (47.73%)	5470867296	5364137540 (98.05%)	5152436402 (94.18%)	70464 (0.00%)	2607256103 (47.66%)
Cj-FLe-3	7477173000	7320092550 (97.90%)	7024662210 (93.95%)	96171 (0.00%)	3582950165 (47.92%)	7414681949	7265160983 (97.98%)	6974798900 (94.07%)	95336 (0.00%)	3548647984 (47.86%)
Cj-ULe-1	6805974900	6679760643 (98.15%)	6428682285 (94.46%)	88331 (0.00%)	3326592400 (48.88%)	6724409692	6605342849 (98.23%)	6359843569 (94.58%)	87146 (0.00%)	3283419693 (48.83%)
Cj-ULe-2	6380562000	6250501961 (97.96%)	5998977436 (94.02%)	83101 (0.00%)	3035739172 (47.58%)	6311275101	6188102739 (98.05%)	5941573444 (94.14%)	82100 (0.00%)	2999563503 (47.53%)
Cj-ULe-3	8506632000	8338525172 (98.02%)	8012749814 (94.19%)	110678 (0.00%)	4179586638 (49.13%)	8420694674	8261252470 (98.11%)	7941450170 (94.31%)	109491 (0.00%)	4134063754 (49.09%)
