# Supplementary material for: The NAC Transcription Factors CjNAC43 and CjNAC54 Act as Positive Regulators of Leaf Senescence in Clerodendrum japonicum
Source: Int J Mol Sci. 2025 Dec 22;27(1):133. doi: 10.3390/ijms27010133 (PMC12785693; doi:10.3390/ijms27010133)
Supplement: Supplementary file 1 [file ijms-27-00133-s001.zip › Table S3 Read mapping statistics against the Clerodendrum japonicum full-length transcriptome reference.pdf]

**Table S3.** Read mapping statistics against the Clerodendrum japonicum full-length transcriptome reference.

| Sample | Total_Genes | Sequenced_Total_Genes(%) |
|---|---|---|
| All | 31366 | 31197 (99.46%) |
| *Cj*-FLe-1 | 31366 | 30507 (97.26%) |
| *Cj*-FLe-2 | 31366 | 30325 (96.68%) |
| *Cj*-FLe-3 | 31366 | 30464 (97.12%) |
| *Cj*-ULe-1 | 31366 | 30500 (97.24%) |
| *Cj*-ULe-2 | 31366 | 30539 (97.36%) |
| Cj-ULe-3 | 31366 | 30624 (97.63%) |
