# Supplementary material for: The NAC Transcription Factors CjNAC43 and CjNAC54 Act as Positive Regulators of Leaf Senescence in Clerodendrum japonicum
Source: Int J Mol Sci. 2025 Dec 22;27(1):133. doi: 10.3390/ijms27010133 (PMC12785693; doi:10.3390/ijms27010133)
Supplement: Supplementary file 1 [file ijms-27-00133-s001.zip › Table S4 List of up-regulated differentially expressed genes (DEGs) in Fle compared to Ule.pdf]

Table S4. List of up-regulated differentially expressed genes (DEGs) in Fle compared to Ule.

Gene name	Cj-FLe	Cj-ULe	Description	log_2
Isoform0010737	0.14	5.60	elongation factor 1-alpha	5.32
Isoform0024995	0.30	8.23	30S ribosomal protein S11, chloroplastic	4.77
Isoform0024489	0.14	2.72	Metal transporter Nramp5	4.28
Isoform0025216	0.63	9.59	Beta-galactosidase 15	3.92
Isoform0029906	0.04	0.58	photosystem II CP47 reaction center-like protein	3.63
Isoform0017812	5.40	63.06	lectin	3.54
Isoform0022912	0.99	10.75	BnaUnng00830D	3.44
Isoform0022369	3.48	34.00	ORF64c, partial	3.28
Isoform0005286	0.09	0.91	acyltransferase-like protein At3g26840, chloroplastic	3.28
Isoform0024254	1.77	16.01	unknow protein	3.17
Isoform0024296	0.37	3.10	Ribulose bisphosphate carboxylase large chain	3.05
Isoform0025573	0.13	1.10	ATP synthase subunit beta, chloroplastic, partial	3.01
Isoform0022979	2.53	19.46	cytochrome b559 subunit alpha	2.93
Isoform0003688	0.10	0.74	acyltransferase-like protein At3g26840, chloroplastic	2.85
Isoform0015873	0.32	2.29	ribulose-1,5-bisphosphate carboxylase/oxygenase large subunit, partial	2.84
Isoform0024146	1.84	13.02	photosystem II protein D1	2.82
Isoform0022412	1.63	11.26	photosystem II protein D1	2.78
Isoform0009324	0.25	1.60	ribulose-1,5-bisphosphate carboxylase/oxygenase large subunit	2.67
Isoform0003063	0.53	3.38	ribulose-1,5-bisphosphate carboxylase/oxygenase large subunit	2.67
Isoform0000492	0.17	1.07	acetyl-CoA carboxylase carboxyltransferase beta subunit	2.65

Note:This table lists the top 20 genes with the greatest up-regulation differences between mature and early senescent leaves of *Clerodendrum japonicum*.
