# Supplementary material for: The NAC Transcription Factors CjNAC43 and CjNAC54 Act as Positive Regulators of Leaf Senescence in Clerodendrum japonicum
Source: Int J Mol Sci. 2025 Dec 22;27(1):133. doi: 10.3390/ijms27010133 (PMC12785693; doi:10.3390/ijms27010133)
Supplement: Supplementary file 1 [file ijms-27-00133-s001.zip › Table S5 List of down-regulated differentially expressed genes (DEGs) in Fle compared to Ule.pdf]

Table S5. List of down-regulated differentially expressed genes (DEGs) in Fle compared to Ule.

Gene name	Cj-FLe	Cj-ULe	Description	log_2
Isoform0008413	5.57	0.01	aldehyde dehydrogenase	-9.12
Isoform0024153	19.84	0.03	metallothionein-like protein 1	-9.07
Isoform0018348	0.53	0.00	40S ribosomal protein S3a	-9.06
Isoform0024305	4.39	0.04	metallothionein-like protein 1	-6.66
Isoform0020310	2.61	0.04	MADS-box transcription factor, plant	-5.91
Isoform0024368	6.47	0.13	hypothetical protein CDL12_07580	-5.63
Isoform0001456	3.88	0.09	protein ALP1-like	-5.43
Isoform0007258	0.41	0.01	UPF0481 protein	-5.36
Isoform0023664	23.52	1.00	transmembrane	-4.54
Isoform0024361	7.93	0.40	protein TIFY 10A-like isoform X4	-4.29
Isoform0027655	183.02	9.96	linoleate 13S-lipoxygenase 3-1, chloroplastic-like	-4.19
Isoform0015757	11.38	0.70	MEKK	-4.01
Isoform0011047	24.61	1.71	uncharacterized acetyltransferase At3g50280	-3.84
Isoform0014552	62.89	4.39	MEKK	-3.84
Isoform0020026	37.35	2.86	secoisolariciresinol dehydrogenase-like	-3.70
Isoform0010416	9.52	0.75	allene oxide synthase, chloroplastic	-3.66
Isoform0023099	3.91	0.31	transmembrane	-3.65
Isoform0016310	110.66	9.05	protein TIFY 10b-like isoform X1	-3.61
Isoform0006750	28.64	2.43	cytokinin dehydrogenase 6 isoform X2	-3.55
Isoform0018273	2.94	0.25	embryo defective 1793 family protein	-3.53

Note:This table lists the top 20 genes with the greatest down-regulation differences between mature and early senescent leaves of *Clerodendrum japonicum*.
