# Supplementary material for: The NAC Transcription Factors CjNAC43 and CjNAC54 Act as Positive Regulators of Leaf Senescence in Clerodendrum japonicum
Source: Int J Mol Sci. 2025 Dec 22;27(1):133. doi: 10.3390/ijms27010133 (PMC12785693; doi:10.3390/ijms27010133)
Supplement: Supplementary file 1 [file ijms-27-00133-s001.zip › Table S6 Identified NAC transcription factor genes in the Clerodendrum japonicum transcriptome.pdf]

Table S6. Identified NAC transcription factor genes in the *Clerodendrum japonicum* transcriptome.

Gene Name	Genes ID	Number of amino acids(aa)	Molecular weight(Da)	PI
CjNAC1	*Isoform0001168*	666	74128.86	5.07
CjNAC2	*Isoform0002990*	612	68301.86	5.19
CjNAC3	*Isoform0003409*	636	70769.18	4.92
CjNAC4	*Isoform0003944*	602	67148.45	4.71
CjNAC5	*Isoform0004338*	389	42971.66	4.77
CjNAC6	*Isoform0004528*	488	54326.07	5.52
CjNAC7	*Isoform0004617*	607	67499.53	4.91
CjNAC8	*Isoform0004938*	478	54366.76	7.58
CjNAC9	*Isoform0004950*	481	54663.56	5.30
CjNAC10	*Isoform0005031*	525	59292.97	4.64
CjNAC11	*Isoform0005453*	578	64306.58	4.64
CjNAC12	*Isoform0005480*	461	51470.23	5.42
CjNAC13	*Isoform0005673*	516	58949.93	4.72
CjNAC14	*Isoform0005891*	*549*	*60959.28*	*4.61*
CjNAC15	*Isoform0005954*	488	54212.64	4.60
CjNAC16	*Isoform0006040*	101	11431.21	10.60
CjNAC17	*Isoform0006075*	389	42788.07	4.74
CjNAC18	*Isoform0006079*	533	59589.49	4.68
CjNAC19	*Isoform0006093*	533	59555.46	4.67
CjNAC20	*Isoform0006235*	584	65171.12	4.81
CjNAC21	*Isoform0006734*	587	65178.02	8.72
CjNAC22	*Isoform0007642*	440	49969.11	5.52
CjNAC23	*Isoform0008809*	466	52955.02	5.36
CjNAC24	*Isoform0010933*	424	49019.46	8.43
CjNAC25	*Isoform0011118*	470	52845.44	4.53
CjNAC26	*Isoform0013053*	284	31974.01	8.96
CjNAC27	*Isoform0013059*	412	47275.1	7.01

CjNAC28	*Isoform0014109*	238	27090.75	9.11
CjNAC29	*Isoform0014389*	370	41697.04	9.49
CjNAC30	*Isoform0014462*	258	30029.63	6.67
CjNAC31	*Isoform0014633*	321	35895.81	8.14
CjNAC32	*Isoform0014789*	321	36724.35	9.54
CjNAC33	*Isoform0014799*	371	41928.94	9.36
CjNAC34	*Isoform0015046*	285	32763.91	9.63
CjNAC35	*Isoform0015047*	363	40827.11	8.59
CjNAC36	*Isoform0015250*	330	36891.53	8.87
CjNAC37	*Isoform0015289*	309	34482.46	8.89
CjNAC38	*Isoform0015870*	324	36471.71	9.26
CjNAC39	*Isoform0016032*	357	40381.08	9.10
CjNAC40	*Isoform0016352*	356	40028.80	8.40
CjNAC41	*Isoform0016404*	314	35941.75	8.88
CjNAC42	*Isoform0016770*	285	32170.32	9.69
CjNAC43	*Isoform0016975*	294	33377.05	9.08
CjNAC44	*Isoform0017013*	260	29135.22	9.18
CjNAC45	*Isoform0017642*	267	30666.14	7.70
CjNAC46	*Isoform0017666*	247	28253.93	9.62
CjNAC47	*Isoform0017934*	260	29165.79	9.63
CjNAC48	*Isoform0018006*	247	28253.93	9.62
CjNAC49	*Isoform0018210*	172	19860.99	5.47
CjNAC50	*Isoform0018456*	232	26774.25	6.45
CjNAC51	*Isoform0019529*	262	30100.80	9.70
CjNAC52	*Isoform0019666*	255	28919.12	9.59
CjNAC53	*Isoform0020301*	273	30971.52	9.26
CjNAC54	*Isoform0020609*	205	23068.14	8.45
CjNAC55	*Isoform0020718*	201	23269.62	8.56
CjNAC56	*Isoform0021970*	233	26916.28	9.34

CjNAC57	*Isoform0023040*	195	22437.39	10.11
