# Supplementary material for: The NAC Transcription Factors CjNAC43 and CjNAC54 Act as Positive Regulators of Leaf Senescence in Clerodendrum japonicum
Source: Int J Mol Sci. 2025 Dec 22;27(1):133. doi: 10.3390/ijms27010133 (PMC12785693; doi:10.3390/ijms27010133)
Supplement: Supplementary file 1 [file ijms-27-00133-s001.zip › Table S7 List of differentially expressed NAC genes related to leaf senescence in Clerodendrum japonicu.pdf]

Table S7. List of differentially expressed NAC genes related to leaf senescence in *Clerodendrum japonicum*.

NO.	Gene name	Genes ID	Description	Symbol
1	*CjNAC16*	*Isoform0006040*	NAC domain-containing protein 2-like	NAC081
2	*CjNAC27*	*Isoform0013059*	NAC domain-containing protein 2-like	NAC081
3	*CjNAC30*	*Isoform0014462*	NAC domain-containing protein 2-like	NAC002
4	*CjNAC36*	*Isoform0015250*	NAC domain-containing protein 72-like	JA2L
5	*CjNAC38*	*Isoform0015870*	NAC domain-containing protein 2-like	NAC048
6	*CjNAC43*	*Isoform0016975*	NAC domain-containing protein 2-like	NAC048
7	*CjNAC46*	*Isoform0017666*	domain-containing protein 83	NAC083
8	*CjNAC48*	*Isoform0018006*	domain-containing protein 83	NAC083
9	*CjNAC54*	*Isoform0020609*	NAC domain-containing protein 72-like	JA2L
