# Supplementary material for: The NAC Transcription Factors CjNAC43 and CjNAC54 Act as Positive Regulators of Leaf Senescence in Clerodendrum japonicum
Source: Int J Mol Sci. 2025 Dec 22;27(1):133. doi: 10.3390/ijms27010133 (PMC12785693; doi:10.3390/ijms27010133)
Supplement: Supplementary file 1 [file ijms-27-00133-s001.zip › Table S8 Primers used for vector construction in this study.pdf]

Table S8. Primers used for vector construction in this study.

Gene Name	Forward (5'-3')	Reversed (5'-3')
CjNAC43	GGTCGACGGTATCGATAAGCTTATGCA TTACTTGGTCCGGAAATGC	GGTGATTTCAGCGAATTATCTAGATC AGCGAGCCGGCGCCGTCCCCCG
CjNAC54	GGTCGACGGTATCGATAAGCTTATGCA TGAATACAGACTCTGTGAATC	GGTGATTTCAGCGAATTATCTAGACT AAACCCGAACCCACCCGCATGGG
