# Supplementary material for: The NAC Transcription Factors CjNAC43 and CjNAC54 Act as Positive Regulators of Leaf Senescence in Clerodendrum japonicum
Source: Int J Mol Sci. 2025 Dec 22;27(1):133. doi: 10.3390/ijms27010133 (PMC12785693; doi:10.3390/ijms27010133)
Supplement: Supplementary file 1 [file ijms-27-00133-s001.zip › Table S9 Primers used for qRT-PCR analysis in this study.pdf]

Table S9. Primers used for qRT-PCR analysis in this study.

Gene Name	Forward (5'-3')	Reversed (5'-3')
CjNAC43	AGTGCAGTTACGTCAGATGAG	GCGTCGAGGTACATGAAGTC
CjNAC54	GGTTGGATGACTGGGTCTTATG	CGA AGAGTCGTGGCTGTATTC
CjUBQ-1	GGACTTCCCGAGTTTGAACAGGTG	TGGATGGCAGGCGTATTCATTATGTC
CjPDS-Q	ACTTTGCCTCCCAGAACATCTCTTG	CGGTTTGGCTGGACTATCTACTGC
AtACTIN2	TCTCCCGCTATGTATGTCGC	GTCACGTCCAGCAAGGTCAA
AtSAG113	CTCTGTCGTAACGGCAAAGC	GTCTGCTGATTACATACGGCTTC
AtSGR1	AACCAACTCAATACGCATCACA	CAACAGAACAAAACGCCACTAA
AtABI5	GAGGCGAGGGTGGTGTTG	GATTAGGTTTAGGATTAGTGGGATG
AtPAO	CTTTTCCACAACCCTTTACGC	GCAAGTGTCCATTCTCATCCAA
AtSAG12	AAGCCAAACTAAAATGTCGCC	CCTTCGCAGCCAAAATCG
